# First Description of the Composition and the Functional Capabilities of the Skin Microbial Community Accompanying Severe Scabies Infestation in Humans

**DOI:** 10.3390/microorganisms9050907

**Published:** 2021-04-23

**Authors:** Charlotte Bernigaud, Martha Zakrzewski, Sara Taylor, Pearl M. Swe, Anthony T. Papenfuss, Kadaba S. Sriprakash, Deborah Holt, Olivier Chosidow, Bart J. Currie, Katja Fischer

**Affiliations:** 1 Scabies Laboratory, Infectious Diseases Program, Biology Department, QIMR Berghofer Medical Research Institute, Brisbane 4006, Australia; charlotte.bernigaud@aphp.fr (C.B.); Sara.Taylor@qimrberghofer.edu.au (S.T.); Pearl.Swe-Kay@qimrberghofer.edu.au (P.M.S.); sri.sriprakash@qimrberghofer.edu.au (K.S.S.); 2 APHP, Hôpital Henri-Mondor, Department of Dermatology, Université Paris-Est, 94000 Créteil, France; olivier.chosidow@aphp.fr; 3 Research Group Dynamic, EA7380, Faculté de Santé de Créteil, Ecole Nationale Vétérinaire d’Alfort, USC ANSES, Université Paris-Est Créteil, 94000 Créteil, France; 4 Genetics and Computational Biology Department, QIMR Berghofer Medical Research Institute, Brisbane 4006, Australia; Martha.Zakrzewski@qimrberghofer.edu.au; 5 Bioinformatics Division, The Walter and Eliza Hall Institute of Medical Research, Parkville 3052, Australia; papenfuss@wehi.edu.au; 6 Department of Medical Biology, University of Melbourne, Melbourne 3010, Australia; 7 Menzies School of Health Research, Charles Darwin University, Darwin 0810, Australia; Deborah.Holt@menzies.edu.au (D.H.); Bart.Currie@menzies.edu.au (B.J.C.); 8 Department of Infectious Diseases, Royal Darwin Hospital, Darwin 0810, Australia

**Keywords:** scabies, *Sarcoptes scabiei*, microbiota, microbiome, skin microbiota, skin microbiome, *Acinetobacter*, GAS, *Streptococcus*, metagenomic

## Abstract

Epidemiological studies link *Sarcoptes scabiei* infection and impetigo. Scabies mites can promote *Streptococcus pyogenes* (Group A *Streptococcus*) and *Staphylococcus aureus* infections by breaching the skin barrier and excreting molecules that inhibit host innate immune responses. However, little is known about the composition and the function of the scabies-associated microbiota. Here, high-throughput whole-metagenome sequencing was used to explore the scabies-associated microbiome. Scabies mites including their immediate microenvironments were isolated from two patients with severe scabies in Northern Australia. Two ~45–50 million paired-end reads Illumina libraries were generated of which ~2 (5.1%) and 0.7 million (1.3%) microbial reads were filtered out by mapping to human (hg19) and mite draft genomes. Taxonomic profiling revealed a microbial community dominated by the phylum Firmicutes (A: 79% and B: 59%) and genera that comprise *Streptococcus*, *Staphylococcus*, *Acinetobacter,* and *Corynebacterium*. Assembly of the metagenome reads resulted in genome bins representing reference genomes of *Acinetobacter baumannii*, *Streptococcus dysgalactiae* (Group C/G), *Proteus mirablis* and *Staphylococcus aureus*. The contigs contained genes relevant to pathogenicity and antibiotics resistance. Confocal microscopy of a patient skin sample confirmed *A. baumannii*, Streptococci and *S. aureus* in scabies mite gut and faeces and the surrounding skin. The study provides fundamental evidence for the association of opportunistic pathogens with scabies infection.

## 1. Introduction

Scabies is a parasitic skin infection caused by the mite *Sarcoptes scabiei* var. *hominis* in humans. The parasite reproduces in the skin and causes a spectrum of diseases from common scabies, with less than a dozen mites over the entire body, to severe profuse and crusted forms, where the number of infesting mites can reach millions [1,2]. With an estimated 200 million cases annually, scabies causes a significant global disease burden worldwide [3,4]. Scabies occurs globally, but is more prevalent in tropical and subtropical countries, and in overcrowded and socially disadvantaged communities [3,4,5]. For example, among Indigenous communities of northern Australia, the scabies prevalence can be as high as 50% in children and 25% in adults [5,6]. In these communities, scabies has a substantial impact on the health and quality of life of people [3]. Most important, secondary bacterial infections can cause serious complications, in addition to itch-related sleep disturbances, social stigma, school and job absenteeism, and increased poverty resulting from chronic disability [3,7].

The human skin is a complex interface between the host and the environment, forming a barrier to infectious microorganisms and playing an important role between the resident skin microbiota, and the host immune response. The microbiota of healthy skin is comprised of taxonomically and functionally diverse microorganisms, most of which are benign commensals residing in epidermal and sub epidermal layers [8]. Skin microbiota composition is multifaceted, shaped by many factors, and highly specific to each individual, and micro-organisms are distributed on the body surface in physiological and topographical distinct niches [9]. When scabies mites enter the epidermis, the alteration of the skin barrier due to burrowing by the mite and scratching by the host often leads to infections with opportunistic pathogens. The major bacteria causing pyoderma (i.e., infection of the epidermal and subepidermal layers of the skin, also called impetigo or skin sores) are *Streptococcus pyogenes* (Group A *Streptococcus*, GAS) and *Staphylococcus aureus* [10]. Once in the skin, these pathogens can cause invasive and severe infections, which are potentially fatal [11]. Post-infectious sequelae associated with GAS include autoimmune-mediated diseases such as acute post-streptococcal glomerulonephritis and acute rheumatic fever [12], which can cause further chronic disease [12]. The prevalence of these diseases in Australian Indigenous communities are amongst the highest in the world, leading to a long-term burden and considerable morbidity, especially in children [13]. Worldwide, GAS remains in the top ten global causes of mortality with an estimated 500,000 deaths a year [12,14]. In 2015, rheumatic heart disease was estimated to be responsible for 319,400 deaths [15,16]. Additionally, patients with severe forms of scabies (i.e., crusted scabies) often present with *S. aureus* bacteraemia [11], leading to potentially life-threatening sepsis [17,18].

Although previous epidemiological studies have clearly identified a link between the epidermal infestation with *S. scabiei* and impetigo [19,20,21,22,23,24,25,26], the molecular pathways linking bacterial pathogens and the scabies mite are still poorly understood. Recent in vitro molecular studies have shown an intriguing tripartite interaction between scabies mites, associated opportunistic bacteria, and the human host [27,28,29,30]. In particular, scabies mites release several classes of intestinal complement inhibiting proteins that are located into the epidermis [31,32,33,34]. These complement inhibitors have been shown to indirectly promote the growth of various GAS [28] and *S. aureus* [29] clinical isolates. In whole blood bacterial assays, complement inhibitors reduce the opsonisation of the bacteria surfaces and thereby decrease phagocytosis by the neutrophils. Indeed, through this mechanism, the mite itself may play a role in the establishment, proliferation, and transmission of GAS and *S. aureus* associated with scabies.

Little is known about the composition of the mite surrounding skin microbiota during a scabies infestation and its functional capabilities. Additional pathogenic bacteria other than GAS and *S. aureus* could be present. In this study, high-throughput whole metagenome sequencing was employed to explore the scabies-associated microbiome in two human patients with crusted scabies. The aim of the study was to provide a first snapshot of the microbiota associated with scabies infection in humans and of the roles these bacteria play in pathogenesis.

## 2. Materials and Methods

### 2.1. Patients and Sample Collection

The collection of the human patient samples was approved by the Human Research Ethics Committee of the Northern Territory Department of Health and Menzies School of Health Research and Menzies School of Health Research (approval 13-2027). Informed consent was obtained from each patient recruited into the study.

Scabies mites were isolated from skin scrapings collected from two unrelated patients with crusted scabies (Patient A and Patient B) from different regional areas of the Northern Territory, Australia. Patient A was a 26-year-old female and Patient B was a 54-year-old male. Over 20 years, both patients had presented with recurrent crusted scabies on multiple occasions. Patient B was one of the two original patients with documented clinical and in vitro ivermectin resistance following prior multiple intermittent therapy with ivermectin [35]. Both patients presented with clinical grade 3 crusted scabies at the time of sampling [2,36]. Neither patient had an identified immunosuppressive comorbidity although both had high serum IgE levels as seen almost universally in crusted scabies [18]. Prior to sampling, the skin was not washed, disinfected or otherwise treated, and no topical or oral antibiotics were administered. Scabies mites (*S. scabiei* var. *hominis*) were individually picked from skin samples collected by scraping the skin of each patient and were pooled. Each skin sample contained between 800 and 1000 mites of all life stages. Pooled isolated mites with their skin microenvironments were not washed to explore both the mite internal and the mite surrounding skin microbiomes.

### 2.2. DNA Extraction and Whole Genome Sequencing

DNA from the mites and their skin environment was extracted from each sample using a Blood and Cell Culture DNA Kit (Qiagen, Chadstone, Victoria, Australia) and a modified procedure, adapted from the manufacturer’s protocol. This protocol is detailed in Mofiz et al. [37,38]. Briefly, samples were immerged in 1 mL of ice-cold lysis buffer (20 mM EDTA, 100 nM NaCl, 1% TritonX-100, 500 mM Guanidine-HCl, 10 mM Tris pH 7.9) and homogenized with stainless steel beads of 2.8 mm diameter at 6800 rpm, 3 cycles, 30 sec per cycle, 30 sec between cycles. The suspension was supplemented with DNase-free RNase A to 0.2 mg/mL and with Proteinase K to 0.8 mg/mL and incubated at 50 °C for 1.5 h. After centrifugation at 4000× *g* for 10 min to pellet insoluble debris, the genomic DNA was isolated on the Qiagen genomic tip as instructed in the manufacturer’s protocol. Two DNA libraries were constructed and 100-base pair paired-end reads were generated on an Illumina HiSeq 2500 instrument at the Australian Genome Research Facility of Melbourne, Victoria, Australia.

### 2.3. Read Pre-Processing

Raw sequencing data was processed for removal of Illumina adaptor sequences, trimmed and quality-based filtered using TRIMMOMATIC software v.0.32 [39] (LEADING:3 TRAILING:3 SLIDINGWINDOW:4:15 MINLEN:30). Paired-end reads were merged using PEAR v.0.9.6 [40]. Scabies mite reads were filtered out by applying BWA-MEM v.0.7.12 [41] using a database of the recently published scabies draft genomes [38,42]. Prior to mapping the sequenced paired reads, the scaffolds of the published draft genomes were examined for microbial contamination by searching for matches to a bacterial, fungal or archaeal NCBI nucleotide sequence using BLAST (>80% nucleotide identity and 25% scaffold coverage) [43]. Reads that did not match the scabies genomes were subsequently mapped to the human assembly GRCh37 (Hg19) using BWA-MEM v.0.7.12 to identify, and remove human host genome contamination.

### 2.4. Whole Metagenome Shotgun Profiling and Binning

The bacterial taxonomic composition and community abundances of the samples were determined using the software KRAKEN v.0.10.5 [44]. The protein alignment tool DIAMOND was applied with an e-value of 10^−5^ to search for sequence matches in the NCBI protein reference database (downloaded 18 March 2016) and to identify the fungal taxonomic composition and abundances [45]. The read sequences were assigned to the taxonomic lowest common ancestor (LCA) using hits whose scores lie within 10% of the best score. The taxonomic assignments were visualised in Krona charts [46]. Reads were assembled using metaspades v3.12 with automatic k-mer sizes adjustment [47]. Metabat2.12.1 was used for binning [48] and checkM v.1.0.14 to assign the bins in taxonomic hierarchy [49]. Contigs in each bin were searched for matches to reference nucleotide sequences in NCBI using BLAST. Genes were predicted and translated into proteins using MetaGeneMark [50].

### 2.5. Bacterial Strain Variation Analysis

Four of the identified bacteria species, which had a predicted assembled completeness of >92%, were selected to be defined at the strain level. The tool PanPhlAn v1.2.2.5 [51] was applied using reference genomes from NCBI (ftp://ftp.ncbi.nlm.nih.gov/genomes/all/GCF) for *Acinetobacter baumannii* (downloaded 10/01/21), *S. dysgalactiae* (25/02/19), *S. aureus* (25/02/19) and *Proteus mirablis* (26/02/19). PanPhlAn profiles different reference strains of a species according to their gene content, which are used as a reference to identify the strain in a metagenomic data. Multi-locus sequence typing (MLST) analysis was done using PubMLST (https://pubmlst.org).

### 2.6. Virulence Factors, Antibiotic Resistance, Degradome and Metabolic Pathway Analysis of the Bacterial Communities

Microbial contigs were searched for matches to the virulence gene sequences obtained from the Virulence Factor Database (VFDB) (downloaded 18/02/18) using BLAST [52]. In addition, metagenome reads were mapped against the virulence factor gene *emm* of *S. pyogenes* (gb|NP_608047) using BWA-MEM [41]. Antibiotic resistance in the assembled metagenome was analysed using the Comprehensive Antibiotic Resistance Database (CARD) (version 3.0.1). SCCmecFinder was used to identify the type of Staphylococcal cassette chromosome mec [53]. The degradome was analysed using the MEROPS database (Release 12.1) by searching for matching genes annotated on the metagenome-assembled genomes using BLAST [54]. Genes and contigs were mapped to KEGG Metabolic pathways (https://www.kegg.jp/) using BLAST.

### 2.7. Localisation of A. baumannii, S. pyogenes, S. dysgalactiae, and S. aureus in the Scabies Mite and in the Surrounding Human Skin

The bacteria of interest were searched and localised within the scabies mites and in the surrounding skin using skin sections obtained from Patient B probed with bacteria-specific antibodies, i.e., *A. baumannii* polyclonal anti-rabbit antibodies (Life span Biosciences, Seattle, WA, USA), *S. pyogenes* polyclonal anti-goat antibodies (My BioSource, San Diego, CA, USA), and *S. aureus* polyclonal anti-rabbit antibodies (My BioSource, San Diego, CA, USA). Goat *Streptococcus* Group A polyclonal antibody (MBS535099, My BioSource, San Diego, CA, USA) was used to localise both *S. pyogenes* and *S. dysgalactiae*.

#### 2.7.1. Testing of the Specificity of Primary Antibodies

Primary antibodies were tested using reference strains (*A. baumannii* ATCC19606, *S. pyogenes* 2031 Type strain emm1, *S. dysgalactiae* NS3396 emm-type STG-480, and *S. aureus* MSSA CC75 M5) grown in Tryptic Soy Broth (Oxoid). Using cavity well slides (Proscitech), 20 µL volumes of bacterial cultures at an OD600 0.1 were incubated on the slide at 37 °C for 24 h, with *A. baumannii* and *S. aureus* incubated aerobically, and *S. pyogenes* and *S. dysgalactiae* slides incubated in 5% CO_2_. After incubation, the slides were heat fixed, then further fixed in 100% methanol for 1 min. After fixation, the staining protocol was carried out as previously outlined, and controls were included by staining each bacterial strain with all three primary antibodies to ensure that there was no cross-reactivity between each strain. Using the Zeiss 780-NLO (North Ryde, New South Wales, Australia) confocal microscope no cross-reactivity was seen, and staining of the appropriate bacteria with the appropriate antibody was demonstrated (Supplementary File, Figure S1).

#### 2.7.2. Localisation of the Bacteria of Interest

To localise mite gut tissue, adjacent serial sections were probed with anti-human Immunoglobulin G (IgG), which is known to be ingested by the mite [55]. Adjacent serial sections were also probed with pre-immune mouse serum as a negative control. Paraffin blocks of 5 mm^3^ skin tissues were used to cut 4 µm sections and coated on X-Tra™ (Leica Biosystems, Wetzlar, Germany) glass slides [56]. The slides were dried at 37 °C for 3 h and dewaxed in xylene followed by graded ethanol. All the incubation steps were done at room temperature (RT) in a humidifier chamber and all the washes were 3× of 5 min with Tris Buffered Saline (TBS pH 7.6). Non-specific protein binding was blocked in 2% BSA in PBS (pH 7.4) for 30 min at RT. The primary antibodies were diluted to 1:500 in 1% BSA + PBS to a final concentration of 6 µg/mL *A. baumannii* antibodies, 1 µg/mL of *S. pyogenes* antibodies, and 8 µg/mL of *S. aureus* antibodies. Primary antibodies were incubated overnight at 4 °C in a humidified box. The following morning, primary antibodies were decanted, and the slides were washed in 3× changes of TBS-T for 5 min. After washing the slides exposed to *A. baumannii* and *S. aureus* antibodies were incubated with secondary Alexafluor 555-labelled donkey anti-rabbit antibodies (Invitrogen, Waltham, MA, USA) diluted 1:1000 (2 µg/mL) in PBS, and the slides exposed to *S. pyogenes* antibodies were incubated with Alexafluor 488-labelled secondary donkey anti-goat antibodies (Invitrogen) diluted to 1:1000 (2 µg/mL). Secondary antibody incubation was for 2 h at RT in a dark humidified box. The secondary antibodies were decanted and the slides washed 3× in PBS for 5 min. The slides were incubated in the dark for 10 min in 1µg/mL of DAPI stain (Sigma Aldrich). The slides were mounted in fluromount aqueous mounting media (Sigma Aldrich) and kept in the dark for the coverslip to set. The slides were imaged using a Zeiss 780-NLO confocal microscope and analysed using Image J software.

An additional serial tissue section was either standard Hematoxylin/eosin (H&E)-stained, to visualise the mite morphology, or probed with an anti-human IgG antibody to highlight the mite digestive system [55]. For the latter, endogenous peroxidase activity was quenched using 5% H_2_O_2_ for 5 min. Slides were washed with 3 rinses of TBS for 5 min, blocked with rabbit serum (1:10 dilution in PBS) for 30 min at RT and washed with 3 rinses of TBS for 5 min. Slides were incubated with horseradish peroxidase (HRP) labelled anti-human IgG (Sigma Aldrich, St. Louis, MO, USA) for 1 h at RT, followed by a wash with 3 washes in TBS. Nova-RED substrate (VECTOR, California, USA) was added to initiate the chromogenic reaction and was stopped by immersion in deionised water for 3 min at RT. The slides were counterstained with haematoxylin for 1 min, dehydrated in graded ethanol, cleared in xylene and mounted with DPX histology slide mounting medium (Sigma Aldrich, St. Louis, MO, USA). Slides were visualised using an Aperio XT Scanscope (Leica Biosystems, Wetzlar, Germany) slide scanner at 40× magnification and analysed using eSlide manager and ImageScope viewing software (Leica Biosystems, Wetzlar, Germany).

## 3. Results

### 3.1. Pipeline

**Figure 1:**
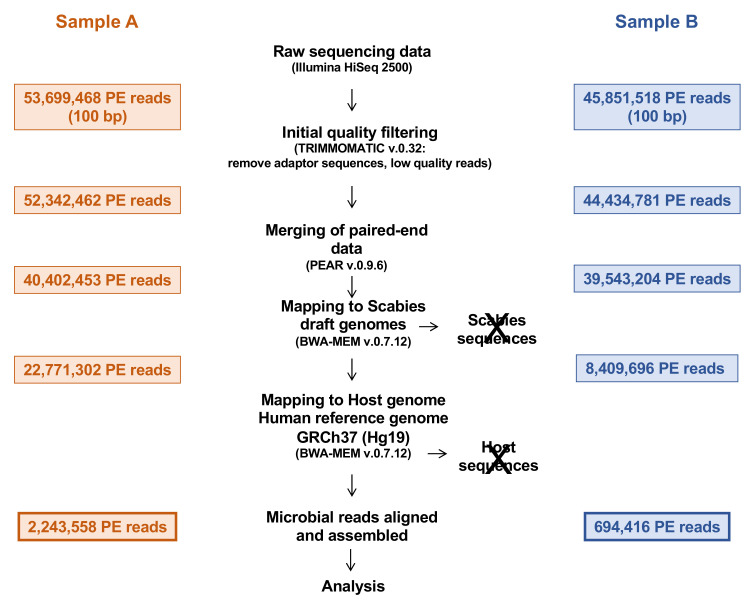
The bioinformatic pipeline illustrating the different steps and the obtained read counts.

**Figure 2:**
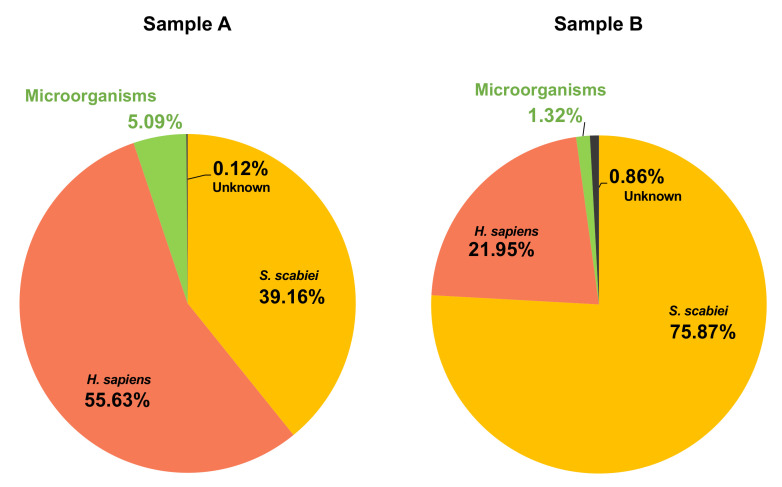
The repartition of the reads sequenced from each patient sample (Patient A and B). Two ~45 to 50 million paired-end read Illumina libraries were generated, of which ~2 million (5.09%) for Patient A and 0.7 million (1.32%) for Patient B were attributed to microbial reads. The other reads were corresponding to human genome (*H. sapiens*) or mite genome (*S. scabiei*) sequences. A small proportion could not be attributed (unknown).

The bioinformatic pipeline illustrating the steps and read counts is shown in Figure 1. Two ~45 to 50 million paired-end read Illumina libraries were generated, of which ~2 million (5.09%) and 0.7 million (1.32%) microbial reads were filtered out by mapping to the human genome (hg19) and the available draft mite genomes [38,42] (Figure 1 and Figure 2).

### 3.2. Microbial Communities: Taxonomic Composition of the Metagenome

**Figure 3:**
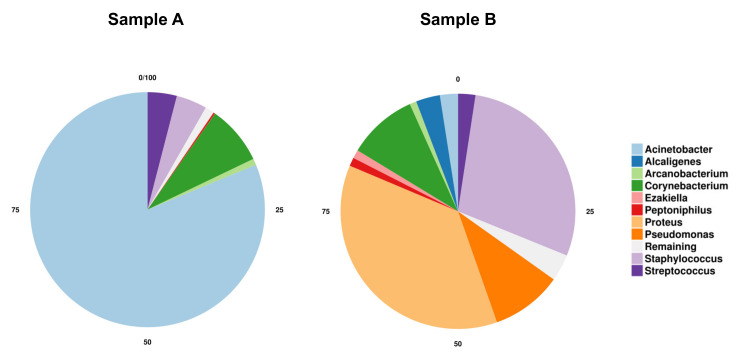
Distribution of the Top 10 most abundant taxa found in samples from Patients A and B at the genus level. The most abundant genera in Patient A sample comprise *Acinetobacter* in light blue (81.2%), *Corynebacterium* in dark green (8.1%), *Staphylococcus* in light purple (4.2%), and *Streptococcus* in dark purple (4%). The most abundant genera in Patient B sample comprise *Proteus* in light orange (36.7%), *Staphylococcus* in light purple (28.7%), followed by *Pseudomonas* in dark orange (9.8%) and *Corynebacterium* in dark green (9.6%).

Taxonomic profiling revealed a complex bacterial community in the samples derived from both patients. At the phyla level, Proteobacteria, Firmicutes and Actinobacteria comprised about 99% of all microbial reads, with the community being dominated by the phylum Firmicutes (Patient A: 79% and Patient B: 59%). The phylum Actinobacteria (Patient A: 9.4% and Patient B: 11.9%), which is known to be most prevalent in normal skin [9], was underrepresented in both patient samples. The pie charts in Figure 3 show differences in the bacterial communities’ composition, indicating the 10 most abundant genera across the two patients. The most abundant genera in the skin sample from Patient A were *Acinetobacter* (81.2%), *Corynebacterium* (8.1%), *Staphylococcus* (4.2%) and *Streptococcus* (4%). In contrast, the microbiome sampled from Patient B was predominantly composed of *Proteus* (36.7%), *Staphylococcus* (28.7%), followed by *Pseudomonas* (9.8%) and *Corynebacterium* (9.6%) at the genus level. In the samples from both patients, the most abundant bacteria are known skin pathobionts that have disease-causing potential. Table 1 presents the most abundant species in both patients.

Eukaryotic fungi were among the rare taxa with less than 0.01% of all classified reads. The most prevalent eukaryotic genus was *Malassezia*. Archaea were absent. Whole-genome shotgun metagenomics allowed the exploration of viral DNA present, which were mainly phages of *S. pyogenes* found in the sample from Patient B and *Acinetobacter* phages in the samples from both patients.

### 3.3. Assembly and Strain-Level Identification of Two Complete Bacterial Genomes from the Scabies-Associated Microbiome of Patient A

Strain-level identification using known strains as a reference was carried out with PanPhlAn tool and assembly was further assessed using alignment dot plots (Supplementary File, Figure S2A). Taxonomic profiling of the metagenomics reads revealed that *A. baumannii* (family *Moraxellaceae*) dominated the microbial community in Patient A. Using hierarchical clustering of the pan genome profile indicated that the strain in Patient A was related to *A. baumannii* J9 (NZ_CP041587.1). Mapping of the metagenome reads obtained from Patient A to the *A. baumannii* J9 genome revealed a high degree of sequence identity with only four coding genes showing sequence differences (Table 2). One of the genes encodes the virulence factor trimeric autotransporter adhesin (Ata), which matched in 2254 of 2258 amino acids. Contig assembly using metaspades and binning analysis combined with a lineage assessment of the bins using checkM indicated similarities of a metagenome-assembled genome (MAG-2) to the *Moraxellaceae* family with a high sequence completeness (100%) and no contamination or strain heterogeneity (0%). In total, the *Moraxellaceae*-related metagenome-assembled genome consisted of 38 contigs with 3608 predicted genes based on metagenemark. Similarity searches using BLAST to the NCBI nucleotide database confirmed that the contigs were highly similar to *A. baumannii* J9 as well as *A. baumannii* 29FS20 (NZ_CP044519.1) (Supplementary File, Table S1).

MLST analysis indicated that all marker genes were present in the metagenome MAG-2 in sample A and was classified as ST-49 or ST-128 based on Pasteur or Oxford marker genes schemes, respectively. A recent publication reported a similar classification of *A. baumannii* sequences [57].

Further metagenome binning identified a metagenome-assembled genome (MAG-3) containing 149 contigs and 2003 predicted genes. Similarity analysis of the contigs indicated a close relationship to *S. dysgalactiae* and checkM predictions estimated about 97% completeness of the metagenome-assembled genome, which was confirmed using alignment dot plots (Supplementary File, Figure S2B). The MLST type 17 (ST-17) was predicted based on seven housekeeping genes (*atoB*, *gki*, *gtr*, *murl*, *mutS*, *recP*, *xpt*) [58]. Strain analysis of the metagenome read sequences in sample A confirmed the presence of *S. dysgalactiae* subsp. *equisimilis*, which typically encode 1878–2223 genes.

### 3.4. Metagenome-Assembled Genomes in the Scabies-Associated Microbiome of Patient B

Strain-level identification was carried out using PanPhlAn tool and genome coverage was assessed using alignment dot plots (Supplementary File, Figure S2C,D). Binning the metagenomic contigs resulted in a metagenome-assembled genome (MAG-1) consisting of 114 contigs and encoding 2455 genes. Quality assessment of this metagenome-assembled genome using checkM indicated a 97% genome completeness and 0.8% contamination based on markers identified in the genus *Staphylococcus*. Most contig sequences within this bin matched the genomes of *S. aureus* subsp. aureus MW2 (BA000033.2, 2632 genes) and *S. aureus* strain MSSA476 (BX571857.1, 2598 genes). Using SCCmecFinder, two SCCmec cassettes were identified, which were predicted as SCCmec_type_I (1B) (gb|AB033763.2) and SCCmec_type_IV (2B) (gb|AB063172.2) (Table 3). In addition, the MLST type based on seven housekeeping genes (*arcC*, *aroE*, *glpF*, *gmk*, *pta*, *tpi*, *yqiL*) was type 1 (ST-1) and *spa* Type t127 [59].

A bin of 306 contigs encoding 3456 genes in sample B was similar to *P. mirabilis* BB2000 (CP004022.1, 3455 genes) and *P. mirabilis* strain AOUC-001 (CP015347, 3853 genes). Analysis with checkM revealed 93% completeness and 1% contamination to the class Proteobacteria.

### 3.5. Identification of Bacterial Virulence Factors Genes

#### 3.5.1. Acinetobacter

One major factor contributing to *A. baumannii* species virulence is their capacity to form biofilm. We investigated the presence of biofilm-related genes using the VFDB and NCBI protein database. We identified several virulence factors genes, the outer membrane protein A *OmpA*, chaperon-usher pilus *CsuE*, component system *BfmRS*, tyrosine-protein kinase *ptk*, *pgaB*, poly-ß-(1,6)-N-acetyl glucosamine *PNAG*, extracellular exopolysaccharide *EPS* and partial biofilm-associated protein *Bap*. No contigs and reads were matching the biofilm formation genes *csgA*, *kpsMII* or *fimH*. We identified both integrase *intA-1* and *intA-2* genes. The biofilm-associated protein (Bap) was predicted on the terminal end of two contigs. Bap is a large cell surface protein, which is critical for cell–cell interaction and biofilm formation [60]. The porin OmpA is involved in attachment and invasion to the host epithelial cells, serum resistance, biofilm formation and persistence and induction of apoptosis [60]. The Csu pilus initiates biofilm formation on abiotic surfaces [60]. The two-component system bfmRS regulates the expression of the csu gene.

#### 3.5.2. Streptococcus

Major streptococcal virulence factors were identified which are typical and essential for these invasive pathogens [6]. In the skin metagenome from sample A, *S. dysgalactiae* genes *lmb* and *gfbA* (partial) coding for adhesins, which are virulence factors related to adherence and internalisation to host cells, were present [61]. One gene matched and completely covered the emm (M-protein) from *S. dysgalactiae* (WP_148848339) with an identity of 98.85%. We did not identify the complete emm (M-protein) of *S. pyogenes* in the skin-assembled metagenome from sample A, and only 37 reads mapped to the reference gene M-protein of *S. pyogenes*. Given the low read counts, no further analysis was carried out. Similarly, genes coding for streptococcal secreted factors such as Streptolysins S and O (*SLS*, *SLO*) or streptococcal exotoxins B (SpeB) were not identified with certainty in the assembled contigs and reads data.

#### 3.5.3. Staphylococcus

The *Staphylococcus* metagenome-assembled genome encoded various virulence factors including toxins (haemolysin, enterotoxins), enzymes relevant for adherence and colonization of the bacterium (clumping factor, fibronectin binding proteins), immune evasion (capsule) and iron uptake [62]. Although at a low read coverage of two reads per position, about 86% of the reference gene for the IgG binding protein A precursor (*SpA*) from *S. aureus* was covered by the dataset from sample B. This gene encodes the bacteria surface protein SPA that binds to the Fc region of IgG which disrupts opsonisation and phagocytosis [62]. SPA can also increase pro-inflammatory cytokine levels and was associated with a reduced skin barrier function and increased severity of skin lesion in an atopic dermatitis mouse model and in human studies [63,64]. The sequence of the gene staphylococcal *esxA* was completely recovered in the data obtained from sample B. This gene is coding for the protein EsxA, a virulence factor present in *S. aureus* strains recovered from lesions of psoriatic patients but absent from strains found at unaffected sites [65].

### 3.6. Antibiotic Resistance

The metagenomes harboured various antibiotic resistance genes including mgrA, mecA, (methicillin-resistance), fusC (fusidic acid), tet38 (tetracycline), mepA (tetracycline), ErmC (macrolide, lincosamide, streptogramin B) from *Staphylococcus* sp., tetJ (tatracycline) from *Proteus* sp. and ADC-3 (cephalosporin), ADC-80 (cephalosporin), ADC-6 (cephalosporin), ADC-68 (carbapenem) and adeIJKLMN (multidrug classes), OXA-98 (penam, cephalosporin), abeS (macrolide, aminocoumarin antibiotic), adeFGH (tetracycline, fluoroquinolone antibiotic), ANT(3′′)-Iia (aminoglycoside antibiotic) from *Acinetobacter* sp.

A betalactamase gene was identified in the metagenome-assembled genome of *A. baumannii*. Several antibiotic resistance proteins were identified in the metagenome-assembled genome of *S. aureus* using the CARD database including the antibiotic efflux pumps ArlR and MgrA, fusidic acid inactivation enzyme FusC, MecA (methicillin resistant PBP2), tetracycline resistant protein (tet38) and multidrug export protein MepA and its repressor mepR (Table 4). Partial sequences of the arlS protein, that forms a complex with ArlR, and the signal protein mecR1, which mediates the gene regulation for the methicillin resistance, were predicted.

### 3.7. Degradome

Using the MEROPS database as a reference, genes with proteolytic functions were identified in the above outlined microbial genomes. In the dataset obtained from the sample from Patient A, genes encoding the intramembrane protease rseP, which transmits the outer membrane environmental stress for example due to antibiotics, and S-formylglutathione hydrolase FrmB for detoxification of xenobiotic molecules in *A. baumannii* were found [66]. In the dataset collected from sample B, predominantly proteases from *S. aureus* were identified including collagenases, the beta-lactamase regulator BlaR1, class C beta-lactamase, the extracellular adherence protein homolog EapH2 and a caseinolytic protease (Clp) involved in the degradation of damaged proteins. Furthermore, the sample from Patient B metagenome encoded pitrilysin, which degrades small peptides and contributes for instance to the pathogenicity of *Vibrio vulnificus* through the proteolytic degradation of insulin [67], and mirabilysin from *P. mirabilis*, which is an IgA-degrading metalloprotease [68].

### 3.8. Metabolic Pathways Analysis Using KEGG Pathways

Mapping of the genes and contigs revealed a good representation of three KEGG pathways, i.e., ‘Bacterial invasion’, ‘*Staphylococcus* infection’ and ‘Cationic antimicrobial peptides’ (Supplementary File, Figure S3). Genes corresponding to the ‘Bacterial invasion’ pathway were identified in the metagenomes and were essentially coding for adhesin proteins and fibronectin-binding proteins (e.g., FnBPA, FnBPB, Pfb or Sfb1). Pathogenic bacteria such as Staphylococci and Streptococci are able to adhere to human skin fibroblasts and to invade epithelial cells using a zipper model mechanism. These Gram-positive bacteria express adhesin proteins on their surfaces that interact with extracellular matrix components of the host such as fibronectin, resulting in close apposition of the epithelial cellular membrane around the entering bacteria [69,70,71] (Supplementary File, Figure S3). Genes from the pathway ‘*Staphylococcus* infection’ were identified in both samples from Patient A and Patient B. Most genes were coding for various virulence factors including: enzymes relevant for adherence and colonisation of the bacterium (e.g., clumping factor ClfB, IsdA adhesin, surface protein G SasG, extracellular fibrinogen-binding protein Efb), immune modulating proteins inhibiting complement activation (e.g., staphylococcal complement inhibitor SCIN), opsonisation (e.g., extracellular complement binding proteins Ecb) or neutrophil chemotaxis (e.g., chemotaxis inhibiting protein CHIPS), proteins modulating immune evasion (e.g., IgG binding protein A SpA and the second immunoglobulin-binding protein Sbi), modulating the sensitivity to cationic antimicrobial peptides (such as defensin) by increasing the positive net charge of its cytoplasmic membrane (e.g., VraF, VraG, Dlt, MprF), and expression of toxins (e.g., haemolysins and enterotoxins) and superantigens [62] (Supplementary File, Figure S3). The third pathway that was identified is the ‘Cationic antimicrobial peptides’ pathway. Cationic antimicrobial peptides (CAMPs) are key components of the innate immune response playing an important role in host defence against microbial infection, particularly into the skin. CAMPs are able to weaken the integrity of the bacterial inner and outer membranes and thereby to kill bacterial cells. Therefore, pathogenic bacteria have developed numerous mechanisms to evade the activity of CAMPs. In the *Staphylococcus* metagenome-assembled genome, various resistance mechanisms to CAMPs were found, comprising the capacity to sense the presence of CAMPs (e.g., GraRS), to decrease the attraction or to repulse them via alteration of cell wall and membrane surface charges (e.g., MprF which modifies phosphatidylglycerol with lysyl residues adding positively charged lysine molecules within the *S. aureus* cell membrane and DltABCD operons that D-alanylate teichoic acids and neutralize the negative net charge of the staphylococcal cell wall), to export them with membrane efflux pumps (e.g., VraFG), to degrade them with secreted proteases (e.g., aureolysin Aur, staphylokinase) and finally to neutralize them by sequestration and external trapping mechanisms [72] (Supplementary File, Figure S3).

### 3.9. A. baumannii, S. pyogenes, S. dysgalactiae, and S. aureus Are Present Scabies Mite Gut and in Surrounding Human Skin

**Figure 4:**
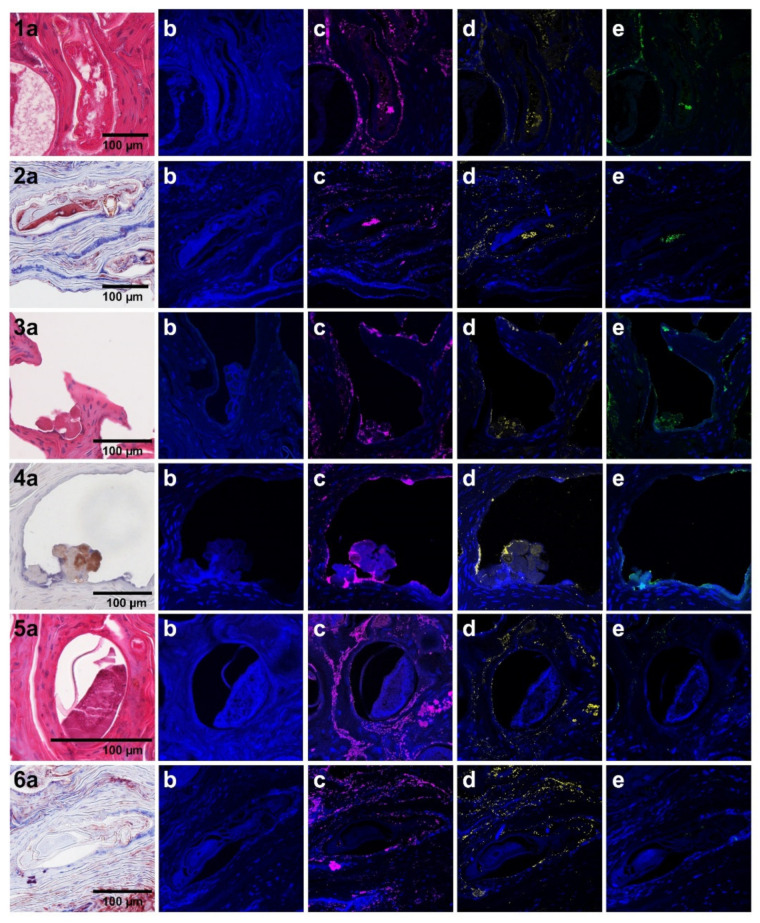
Immunohistological localisation of *A. baumannii, S. pyogenes, S. dysgalactiae* and *S. aureus* in *S. scabiei* var. *hominis*-infested epidermal tissue by confocal microscopy. Series 1–6 are serial histological sections of scabies mite-infested human skin showing localised bacteria. Series 1 and 2 feature adult *S. scabiei* var. *hominis* mites within epidermal burrows; series 3 and 4 show mite faecal pellets; series 5 depicts a laid egg, and series 6 an unlaid egg within an adult female mite. Adjacent section of each series were H&E-stained (**1a**, **3a**, and **5a**) or probed with anti-human IgG as a mite gut marker (**2a**, **4a**, and **6a**). In these sections, red staining indicates antibody binding to protein. Sections in column (**b**) were probed with pre-immune mouse sera (negative control). Sections in columns (**c**–**e**) were probed with antibodies specific for *S. aureus, A. baumannii,* and *S. pyogenes/S. dysgalactiae,* respectively. The scale bar is 100 µm.

We were able to localize *A. baumannii*, *S. pyogenes*, *S. dysgalactiae* and *S. aureus* within the scabies mites and in the surrounding skin using skin sections and probing with bacteria-specific antibodies. Within the scabies mite, the bacteria were present in the mite digestive system (Figure 4, Panel 1C–E and 2C–E) and were released into the epidermal burrows, with the mite faeces (Figure 4, Panel 3C–E and 4C–E). We did not observe any bacteria within intact *S. scabiei* eggs; hence, we postulate that freshly hatched larvae take up the bacteria when feeding.

## 4. Discussion

In this study, a metagenome sequencing approach on the Hi-Seq Illumina platform was employed to investigate the microbial community accompanying severe scabies infection (i.e., crusted scabies), in two human patients residing in northern Australia. By picking the parasites from unwashed and untreated skin samples, the microbial communities residing internally within the mite, and externally on the mite surface and within the skin microenvironment were captured. The microbiome analysis confirmed the presence of pathogenic bacteria of the genera *Streptococcus* and *Staphylococcus* within scabies-infested skin, and identified further associated pathogens such as *A. baumannii*, *Pseudomonas aeruginosa*, and *Proteus mirabilis*.

From the sample of Patient A, we could assemble two bacterial genomes, classified as *A. baumannii* and *S. dysgalactiae* subsp. *equisimilis*. *A. baumannii* is a Gram-negative bacterium that is ubiquitous in nature and a commensal of human skin, present in 20–25% of humans [73]. The bacterium can be pathogenic as it adheres to and invades human host cells causing bacteraemia, wound infections, pneumonia, endocarditis or meningitis [74,75,76,77,78,79]. *A. baumannii* has been identified as an important nosocomial pathogen due to its ability to form biofilms, modulate host cell signalling, induce apoptosis, develop serum resistance, and perform immune evasion [80]. Interestingly, genes that varied between the recently sequenced *A. baumannii* genome J9 and the isolate identified here are relevant in the process of attachment and invasion of human host cells. The autotransporter adhesion (Ata) protein mediates adhesion to the extracellular matrix proteins and virulence in a murine pneumonia model [81]. Experiments with an Ata knockout strain have indicated that *A. baumannii* induces host cell secretion of pro-inflammatory cytokines IL-8 during early and IL-6 during later infection stages and monocytes recruitment [81]. In some pathogens, motility organelles contribute to biofilm formation and host cell adherence. The type IV pili (T4P) is a bacterial appendage ubiquitous within *A.* *baumannii* and composed of a cytoplasmic AAA + ATPase (PilB), an integral membrane protein (PilC) and at least two pilins (PilA). *A. baumannii* was declared the number one priority pathogen for research by the World Health Organisation due to its pathogenicity related to a growing multidrug antibiotic resistance [82]. A mechanism to develop resistance to host immune responses is by prophage-mediated gene transfer. Herein, we have identified two integrases and several transposases of the family IS3 and IS5. The genome also carried virulence factors involved in the uptake of iron and other micronutrients, motility, production of cytotoxic and protection factors, biofilm formation and adhesion to and invasion into human host cells.

Group C and G Streptococci such as *S. dysgalactiae* subsp. *equisimilis* are phylogenetically very similar to *S. pyogenes*. They can be skin commensals or pathogens inducing similar spectrum of illness as caused by GAS, such as wound infections, erysipelas and cellulitis. *S. dysgalactiae* subsp. *equisimilis* is also reported to be associated with life-threatening necrotizing fasciitis and streptococcal toxic shock syndrome [83]. Notably, very low level of GAS is present in the sample from Patient A, which contains a high abundance of *S. dysgalactiae* subsp. *Equisimilis*. GAS and *S. dysgalactiae* subsp. *Equisimilis* are phylogenetically close, compete for the same resources and cause a similar disease spectrum in humans. *S. dysgalatiae* subsp. *Equisimilis* have been reported to produce antimicrobial proteins specifically active against GAS [84] and this may explain the low abundance of GAS in this case.

The data retrieved from the sample from Patient B was dominated by metagenome-assembled genomes related to *S. aureus*. The bacterium *S.* *aureus* is both a frequent commensal of the skin and a common human pathogen, causing skin and soft tissue infections, bacteraemia and osteomyelitis [85,86]. *S.* *aureus* species are characterised by their high resistance against common antibiotics and are prominent pathogens causing persistently high mortality worldwide. The most important laboratory documented resistance is to methicillin, an anti-staphylococcal penicillin that is a surrogate used in laboratory testing for flucloxacillin resistance. Methicillin resistance in *S. aureus* (MRSA) is mediated by the mecA gene, which is embedded in a mobile genetic element termed staphylococcal cassette chromosome mec (SCCmec). SCCmec also includes site-specific recombinase genes (ccrA, crrB) enabling the mobility of the region and is inserted within the orfX gene (RNA methyltransferase) [86]. Infection due to community-associated strains of MRSA was first recognized in Australia in people from remote Indigenous communities of northern Australia [87]. They now have a very high prevalence of MRSA infections, correlated with measures of remoteness and socioeconomic disadvantage. One of the *S. aureus* strains in the sample from Patient B was identified as spa type t127, sequence Type ST-1. This strain, which is methicillin-resistant, has been frequently isolated in pigs in some countries, mostly in Europe, and is considered as the most widespread methicillin-resistant livestock-associated *S. aureus* [88]. Mofiz et al. [37] compared the mite mitochondrial genome data from pig mites (*S. scabiei* var. *suis*) to mites from humans (*S. scabiei* var. *hominis*). In a Single Nucleotide Polymorphism (SNP) analysis of mite gDNA from the same two human samples under investigation here (Patient A and B) and from four pig samples, the authors identified 16 mtDNA haplotypes in total, falling into two broad clades and six distinct haplogroups [37]. Three pig mite haplogroups (seven haplotypes) and three human mite haplogroups (four haplotypes) were identified. In a phylogenetic tree, all pig mite haplogroups clustered closely. Among the three human mite haplogroups, two grouped together, with two Patient A human mite haplotypes (H1_A and H2_A) being essentially clonal, and Patient B haplotype H2_B being very close to these. Between these human and pig mite haplotypes, a sequence divergence of about 600 SNPs was observed. The fourth human mite haplotype (H1_B_REF) however, was very close to the pig mite haplotypes, with only about 80 SNPs difference [37]. This data indicates that some human and pig mites may be very closely related. We could speculate that Patient B may have lived around livestock animals such as pigs and that they could have shared microorganisms. If so, the *S. aureus* strain from Patient B may be zoonotically acquired, possibly from pigs. Indeed, scabies mites could have acted as a vector for transmission of this pathogenic bacterium.

We also found evidence of *P. mirabilis* and *P.* *aeruginosa.* Both microorganisms are Gram-negative, rod-shaped bacteria widely distributed in soil and water. *P. mirabilis* is most frequently involved in infections of the urinary tract [89], and rarely found in the skin, whereas *P.* *aeruginosa* can be involved in skin infections, for example after burn injuries or any cause of rupture of the skin barrier [90].

It is generally accepted that microbiota play an important role in protecting the host from pathogens. Resident microbiota perform this protective function in a number of ways, such as colonisation resistance and modulating the host immune system. Colonisation resistance involves limiting the nutrients and space for initial colonisation of potential pathogens. In addition, the microbiota may modulate the types of immune reaction or secretion of antimicrobial peptides by the host against certain groups of microbes, thereby maintaining homeostasis. Perturbation of the resident skin microbiota (i.e., dysbiosis) during a scabies infestation was demonstrated in vivo in a porcine experimental model [87]. With the onset of scabies mite infection, the healthy resident skin microbiota changed rapidly and dramatically. The main observation was a general reduction in the microbial diversity and a massive rise of pathogenic Staphylococcal species (foremost *S. chromogenes*) [91]. These bacteria are well-known pathogens in pigs, however in healthy porcine skin they are kept at low numbers. We propose that, when epidermal barrier and microbial composition are disrupted, opportunistic pathogens may switch on their virulent traits [92]. At the same time, the burrowing scabies mites release molecules into the skin that interfere with the host innate immune response [31,32,33,34], promoting the growth of opportunistic bacteria [28,29,30,34].

In this study, we sequenced both the mite external (i.e., epidermal microbiota associated with the mite) and the mite internal (intestinal) microbiota. We therefore cannot determine here if some of the identified pathogenic bacteria were originally host skin bacteria, which were amplified due to mite infection, or if the scabies mites may even act as a vector for transmission of some of these pathogenic bacteria. We were limited to investigating samples from crusts from scabies patients and were not in the position to generate a patient-specific ‘healthy skin’ control dataset. Consequently, we are not able to establish whether the opportunistic pathogens were present as a part of the normal skin flora. Arthropods have their own, distinct microbiota, which play a role in their biology and pathogenicity. Often arthropod microbiota include symbionts, which may provide the host with specific essential nutrients, defence against external factors or which may manipulate reproduction. Disruption or removal of the internal microbiota and endosymbionts in arthropods has been reported to reduce survival, fecundity and growth of arthropods without inhibiting feeding [93,94,95]. Aiming to investigate the internal microbiota of scabies mites, metagenome analysis of pig mites (*S. scabiei* var. *suis*) has detected the presence of opportunistic pathogen, *Klebsiella pneumonia* and a probable bacterial symbiont, namely *Streptomyces* [96]. The findings were confirmed histologically with FISH analysis [96]. This study highlighted that the scabies mite indeed may act as a vector for transfer of opportunistic pathogens. Future exploration into the internal microbiome will give a better understanding of the scabies mite biology.

Bouvresse et al. described the presence of *A. baumannii* in human head lice (*Pediculus humanus capitis*), present in the hair of elementary schoolchildren in Paris, France. Using molecular detection methods, the authors were able to detect *A. baumannii* DNA in 33% of the target population (95 positives samples out of 288, 74 schools from 2008 to 2009) [97]. Similar findings were reported from Ethiopia, Thailand, Algeria, Malaysia, and Gabon [98,99,100,101,102]. In addition, the bacterium was also present in body lice (*Pediculus humanus morphotype corporis*) [103]. It will require further studies to clarify whether arthropod hosts, insects or acari, can act as hidden environmental reservoirs for bacteria and whether they can promote the spread of the bacteria between individuals in host communities.

This study is the first to describe the composition of the skin microbial community that accompanies severe scabies infection in humans. We emphasize that this exploratory pilot study provides an exemplary (not representative) picture. While limited to samples from only two patients, the data unveiled the presence of numerous pathogenic bacteria present in the mite-infested skin. This microbiota harbours complex functional capabilities that may be relevant to their invasion of the human host and to enhancing their pathogenicity. Based on these crucial fundamental findings, we are currently conducting a larger amplicon-based study including patients from four different countries with different socio-economic features and climates, to investigate the impact of scabies infection on the associated skin microbiota.

## Figures and Tables

**Table 1:** Relative abundance (%) of the bacterial species found in samples from Patient A and Patient B. Only species present with an abundance >0.01% are shown.

Species	Sample from Patient A	Sample from Patient B
*Acinetobacter_baumannii*	59.77	1.1
*Proteus_mirabilis*	0.01	41.09
*Staphylococcus_aureus*	3.38	23.9
*Streptococcus_dysgalactiae*	5.54	1.88
*Pseudomonas_aeruginosa*	0.00	5.75
*Corynebacterium_striatum*	5.28	0.17
*Corynebacterium_jeikeium*	0.87	5,00
*Staphylococcus_pettenkoferi*	0.02	4.28
*Acinetobacter_nosocomialis*	4.17	0.08
*Alcaligenes_faecalis*	0.00	3.26
*Acinetobacter_pittii*	3.18	0.06
*Corynebacterium_diphtheriae*	2.66	1.55
*Corynebacterium_resistens*	2.65	0.26
*Staphylococcus_argenteus*	2.59	0.1
*Acinetobacter_calcoaceticus*	1.45	0.03
*Arcanobacterium_haemolyticum*	1.41	1.06
*Ezakiella_massiliensis*	0.02	1.3
*Peptoniphilus_harei*	0.34	1.25
*Acinetobacter_soli*	1.23	0.02
*Finegoldia_magna*	1.02	0.3
*Corynebacterium_xerosis*	0.35	0.85
*Corynebacterium_aurimucosum*	0.05	0.68
*Acinetobacter_lactucae*	0.67	0.02
*Staphylococcus_cohnii*	0.00	0.64
*Porphyromonas_asaccharolytica*	0.00	0.6
*Acinetobacter_oleivorans*	0.4	0.01
*Achromobacter_xylosoxidans*	0.00	0.4
*Acinetobacter_sp._ACNIH1*	0.38	0.1
*Peptostreptococcaceae_bacterium_oral_taxon_929*	0.01	0.38
*Corynebacterium_simulans*	0.37	0.13
*Staphylococcus_epidermidis*	0.03	0.36
*Fastidiosipila_sanguinis*	0.35	0.14
*Anaerococcus_mediterraneensis*	0.08	0.32
*Streptococcus_pyogenes*	0.08	0.27
*Corynebacterium_urealyticum*	0.03	0.27
*Corynebacterium_sp._ATCC_6931*	0.07	0.25
*Alcaligenes_aquatilis*	0.00	0.2
*Acinetobacter_sp._FDAARGOS_493*	0.19	0,00
*Staphylococcus_capitis*	0.19	0.09
*Campylobacter_ureolyticus*	0.00	0.18
*Anaerococcus_prevotii*	0.05	0.15
*Acinetobacter_sp._WCHA55*	0.15	0,00
*Corynebacterium_lactis*	0.14	0.08
*Escherichia_coli*	0.00	0.13
*Staphylococcus_haemolyticus*	0.08	0.11
*Acinetobacter_schindleri*	0.01	0.1
*Streptococcus_pseudoporcinus*	0.01	0.1
*Brevibacterium_linens*	0.02	0.09
*Providencia_stuartii*	0.00	0.09
*Corynebacterium_singulare*	0.03	0.08
*Acinetobacter_junii*	0.08	0.01
*Corynebacterium_falsenii*	0.01	0.08
*Staphylococcus_sciuri*	0.01	0.08
*Acinetobacter_sp._NCu2D2*	0.08	0.00
*Salmonella_enterica*	0.00	0.08
*Streptococcus_agalactiae*	0.03	0.06
*Corynebacterium_sphenisci*	0.02	0.05
*Corynebacterium_imitans*	0.01	0.05
*Corynebacterium_segmentosum*	0.01	0.05
*Corynebacterium_sp._2183*	0.01	0.05
*Klebsiella_pneumoniae*	0.01	0.05
*Pseudomonas_chlororaphis*	0.01	0.05
*Tessaracoccus_sp._MarseilleP5995*	0.05	0.01
*Brevibacterium_aurantiacum*	0.02	0.04
*Dermabacter_vaginalis*	0.04	0.02
*Acinetobacter_haemolyticus*	0.04	0.01
*Acinetobacter_sp._ACNIH2*	0.04	0.00
*Acinetobacter_wuhouensis*	0.04	0.00
*Staphylococcus_schweitzeri*	0.02	0.03
*Acidovorax_sp._KKS102*	0.03	0.00
*Acinetobacter_equi*	0.03	0.00
*Acinetobacter_sp._WCHA45*	0.03	0.00
*Acinetobacter_defluvii*	0.02	0.00
*Acinetobacter_ursingii*	0.02	0.00

**Table 2:** Single nucleotide variation in the metagenomic strain using *A. baumannii* J9 as a reference. Ribosomal gene, tRNA genes, transposases, and prophage integrase A were excluded.

SNP Position	Gene in *A. baumannii* J9	Gene Annotation
731653	FK728_00685	Hypothetical protein (blastp: putative pilus assembly protein FilE)
1128536, 1128538, 1128542, 1128545, 1128550, 1128776, 1128791, 1128842, 1128848, 1128854, 1128857, 1128878, 1128888, 1128890, 1130394	FK728_01048 FK728_01049	Adhesin Ata autotransporter (hypothetical protein)
1166479, 1166485, 1166524, 1166527, 1166533, 1166539, 1166548, 1166571, 1166593, 1166602, 1166606, 1166610, 1166617, 1166623, 1166626, 1166662, 1166673, 1166677	FK728_01109	Hypothetical protein (blastp: tape measure protein) tape measure protein
2998436, 2998781, 2998895, 2999189, 2999191, 2999198, 2999200, 2999201, 2999321, 2999468, 2999707, 2999714, 2999813, 3000221, 3000223, 3000230, 3000232, 3000233, 3000353, 3000500, 3000737, 3000739, 3000746, 3000748, 3000749, 3000845, 3000869, 3001361, 3001385, 3001769, 3001771, 3001778, 3001780, 3001781, 3001901, 3002153, 3002285, 3002287, 3002294, 3002296, 3002297, 3002417, 3002564, 3002801, 3002803, 3002810, 3002812, 3002909, 3002933, 3003319, 3003326, 3003328, 3003329	FK728_02809	Biofilm-associated protein

**Table 3:** SCCmec cassettes genes identified in the metagenome-assembled *Staphylococcus aureus* genome. SCCmec, Staphylococcal Cassette Chromosome *mec*; HSP, high scoring pair.

SCCmec Genes	% Identity	Query/HSP Length	Contig	Position in Contig
ccrA2:7:81108:AB096217	100	1350/1350	NODE_170_length_13084_cov_4.268171	10888..12237
ccrB1:1:COL:CP000046	92.31	1626/1625	NODE_48_length_30375_cov_4.414413	13661..15286
ccrA1:1:COL:CP000046	94.37	1350/1350	NODE_48_length_30375_cov_4.414413	15308..16657
subtype-IVa(2B):1:CA05:AB063172	100	1491/1491	NODE_48_length_30375_cov_4.414413	184..1674
mecA:12:AB505628	100	2010/2010	NODE_170_length_13084_cov_4.268171	2473..4482
dmecR1:1:AB033763	100	987/987	NODE_170_length_13084_cov_4.268171	4579..5565
IS1272:3:AM292304	100	1843/1843	NODE_170_length_13084_cov_4.268171	5554..7396
ccrB2:9:JCSC4469:AB097677	99.88	1650/1650	NODE_170_length_13084_cov_4.268171	9238..10887

**Table 4:** List of genes with hits to the CARD database based on similarity searches.

Gene	Reference	Annotation	Identity (%)	Reference Covered (%)
gene_id_37	WP_001283444.1	mgrA	100	100
gene_id_770	YP_001440920.1	mepR	100	92.80576
gene_id_1437	AAV80464.1	tet(38)	99.556	100
gene_id_1460	WP_001033157	fusC	100	100
gene_id_2147	AGC51118.1	mecA 99.701	668	100
gene_id_2148	YP_001245420.1	mecR1	100	41.88034
gene_id_2332	WP_000192137.1	arlR	100	100
gene_id_2333	YP_499945.1	arlS 100.000	383	84.92239

## Data Availability

The data presented in this study are available on request from the corresponding author or Matha Zakrzewski (Martha.Zakrzewski@qimrberghofer.edu.au).

## Supplementary Materials

The following are available online at https://www.mdpi.com/article/10.3390/microorganisms9050907/s1. Figure S1: Testing of the specificity of primary antibodies, Figure S2: Alignment dot plots, Figure S3. Mapping of the genes and contigs to KEGG pathways, Table S1: The 38 contigs of the metagenome-assembled genome MAG-2 blast to *A. baumannii* genomes in the NCBI nucleotide database.
