# Supplementary material for: First Description of the Composition and the Functional Capabilities of the Skin Microbial Community Accompanying Severe Scabies Infestation in Humans
Source: Microorganisms. 2021 Apr 23;9(5):907. doi: 10.3390/microorganisms9050907 (PMC8146700; doi:10.3390/microorganisms9050907)
Supplement: Supplementary file 1 [file microorganisms-09-00907-s001.zip › microorganisms-1166653-supplementary.pdf]

SUPPLEMENTARY APPENDIX

First description of the composition and the functional capabilities of the skin microbial community accompanying severe scabies infection in humans

Charlotte Bernigaud [1,2,3,*], Martha Zakrzewski [4,*], Sara Taylor [1], Pearl M. Swe [1], Anthony T. Papenfuss [5,6], Kadaba S. Sriprakash [1], Deborah Holt [7], Olivier Chosidow [2,3], Bart J. Currie [7,8], and Katja Fischer [1]

[1]Infectious Diseases Program, Biology Department, Scabies Laboratory, QIMR Berghofer Medical Research Institute, Brisbane, Australia

[2]APHP, Hôpital Henri-Mondor, Department of Dermatology, Université Paris-Est, Créteil, France

[3]Research Group Dynamic, EA7380, Faculté de Santé de Créteil, Ecole Nationale Vétérinaire d'Alfort, USC ANSES, Université Paris-Est Créteil, Créteil, France

[4]Genetics and Computational Biology Department, QIMR Berghofer Medical Research Institute, Brisbane, Australia

[5]Bioinformatics Division, The Walter and Eliza Hall Institute of Medical Research, Parkville, Victoria, Australia

[6]Department of Medical Biology, University of Melbourne, Melbourne, Victoria, Australia

[7]Menzies School of Health Research, Charles Darwin University, Darwin, Australia

[8]Department of Infectious Diseases, Royal Darwin Hospital, Darwin, Australia

*C. Bernigaud and M. Zakrzewski contributed equally to this article.

SUPPLEMENTARY APPENDIX

CONTENTS

MATERIALS AND METHODS ……………………………………………………..……..... 3

1.Localisation of *A. baumannii*, *S. pyogenes*, *S. dysgalactiae*, and *S. aureus* in the scabies mite and in the surrounding human skin…...……………….……………………….…. 3

Figure S1. Testing of the specificity of primary antibodies.…………………….…..…… 3

RESULTS ……………………………………………………………………………... 4

*1.*Assembly and strain-level identification of two complete bacterial genomes from the scabies-associated microbiome of Patient A……………………………………………... 4

Figure S2. Alignment dot plots …………..………………………………...……………. 4

Table S1. The 38 contigs of the metagenome-assembled genome MAG-2 blast to *A. baumannii* genomes in the NCBI nucleotide database.……………………….…………… 9

*2.*Metabolic pathways analysis using KEGG pathways………………..…………….10

Figure S3. Mapping of the genes and contigs to KEGG pathways…………..………. 10

MATERIALS AND METHODS

1. Localisation of *A. baumannii*, *S. pyogenes*, *S. dysgalactiae*, and *S. aureus* in the scabies mite and in the surrounding human skin

Figure S1. Testing of the specificity of primary antibodies.

Primary antibodies were tested using reference strains, *i.e. S. aureus* MSSA CC75 M (a)*, A. baumannii* ATCC19606 (b)*, S. pyogenes* 2031 Type strain emm1 (c), *S. agalactiae 18RS21* Type II (d), and *S. dysgalactiae* NS3396 emm-type STG-480 (e). Each bacterial strain was stained with all three primary antibodies, *i.e. S. aureus* polyclonal anti-rabbit antibodies (My BioSource) (a), *A. baumannii* polyclonal anti-rabbit antibodies (Life span Biosciences) (b), and *S. pyogenes* polyclonal anti-goat antibodies (My BioSource) (c-e); to ensure that there was no cross-reactivity between each strain. Using the Zeiss 780-NLO confocal microscope no cross-reactivity was seen, and staining of the appropriate bacteria with the appropriate antibody was demonstrated. However, as can be seen in figures 7c-e the Streptococcal antibody was not species specific.

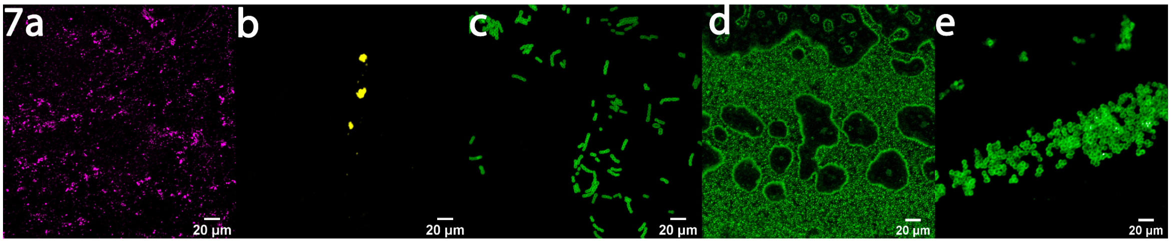

RESULTS

1. Assembly and strain-level identification of two complete bacterial genomes from the scabies-associated microbiome of Patient A

Figure S2. Alignment dot plots.

Reference genomes were selected using the strain-level identification tool PanPhlAn. The figure shows the plots of mapping the metagenome-assembled genome (MAG) contigs onto reference sequences. Each reference sequence is positioned on the x axis, the contigs for the MAG are listed on the Y axis. Each red line represents a contig from a MAG and illustrates the position and orientation of the contig on the reference genome. Plot were generated using MUMMER (version 3.5).

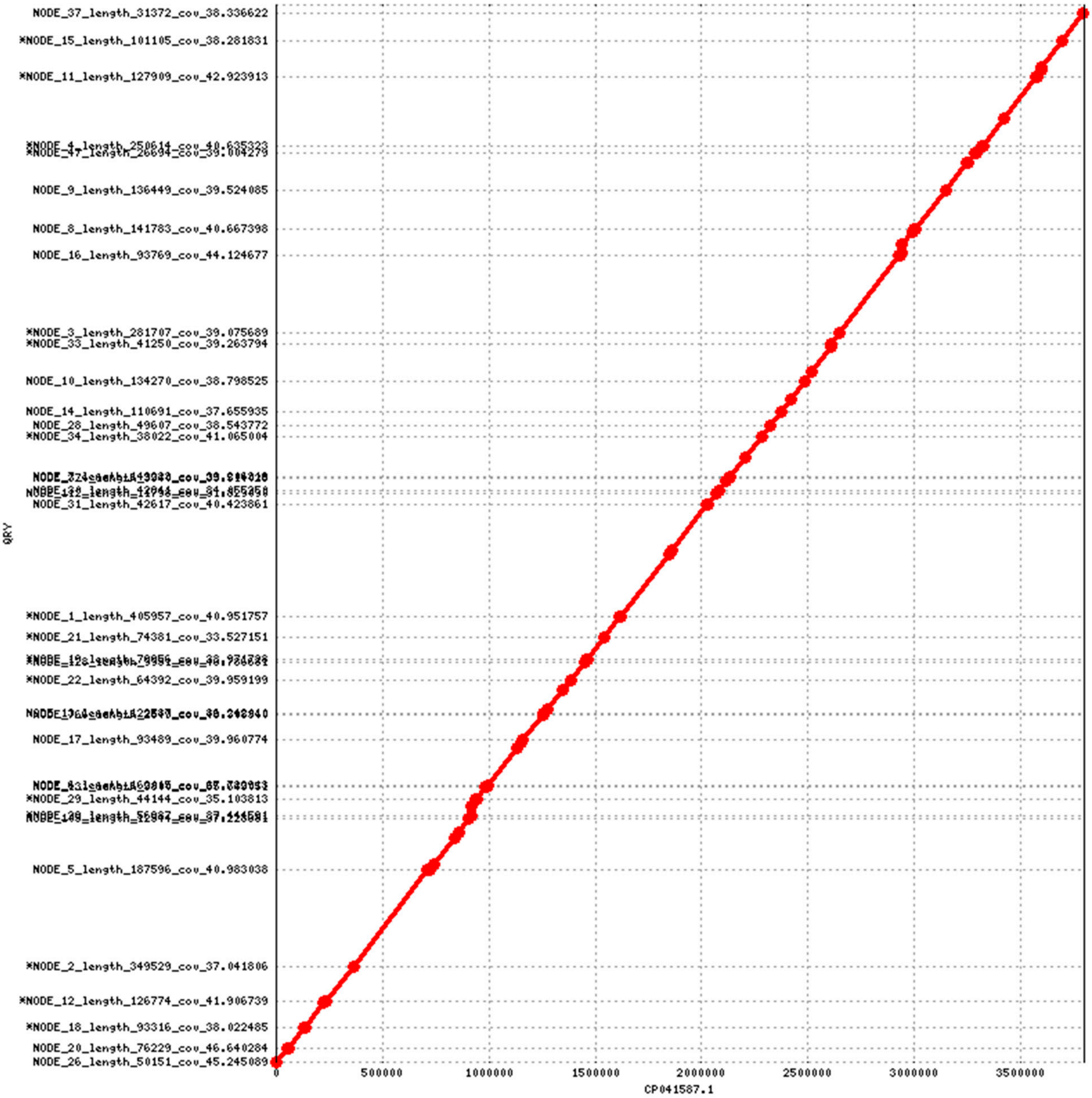

A. **Mummer plot of the MAG-2 onto reference sequence of *Acinetobacter baumannii* J9 (NZ_CP041587.1) (sample from Patient A).**

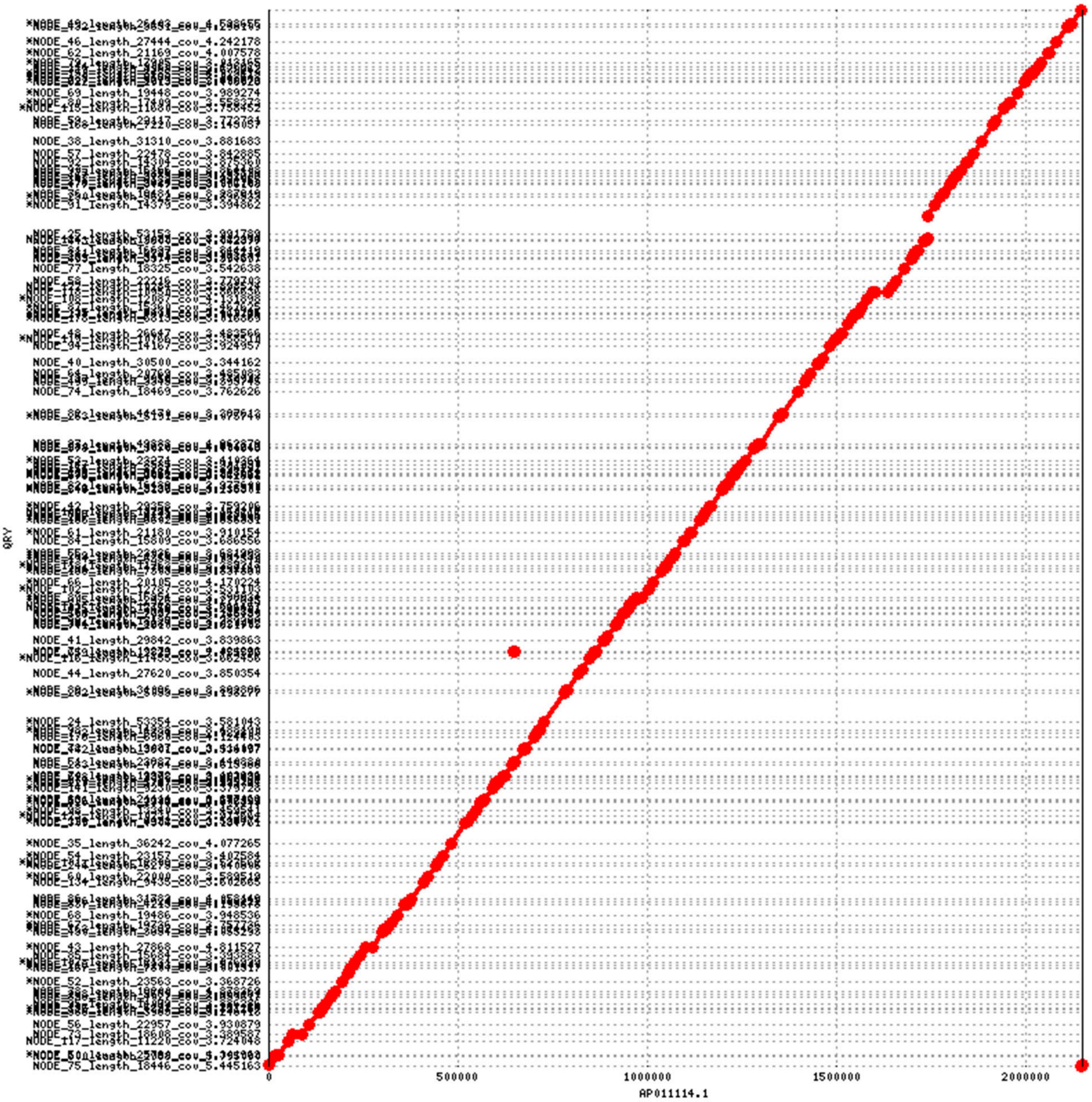

B. Mummer plot of the MAG-3 onto reference sequence of *Streptococcus dysgalactiae* subsp. equisimilis (AP011114.1 RE378) (sample from Patient B)

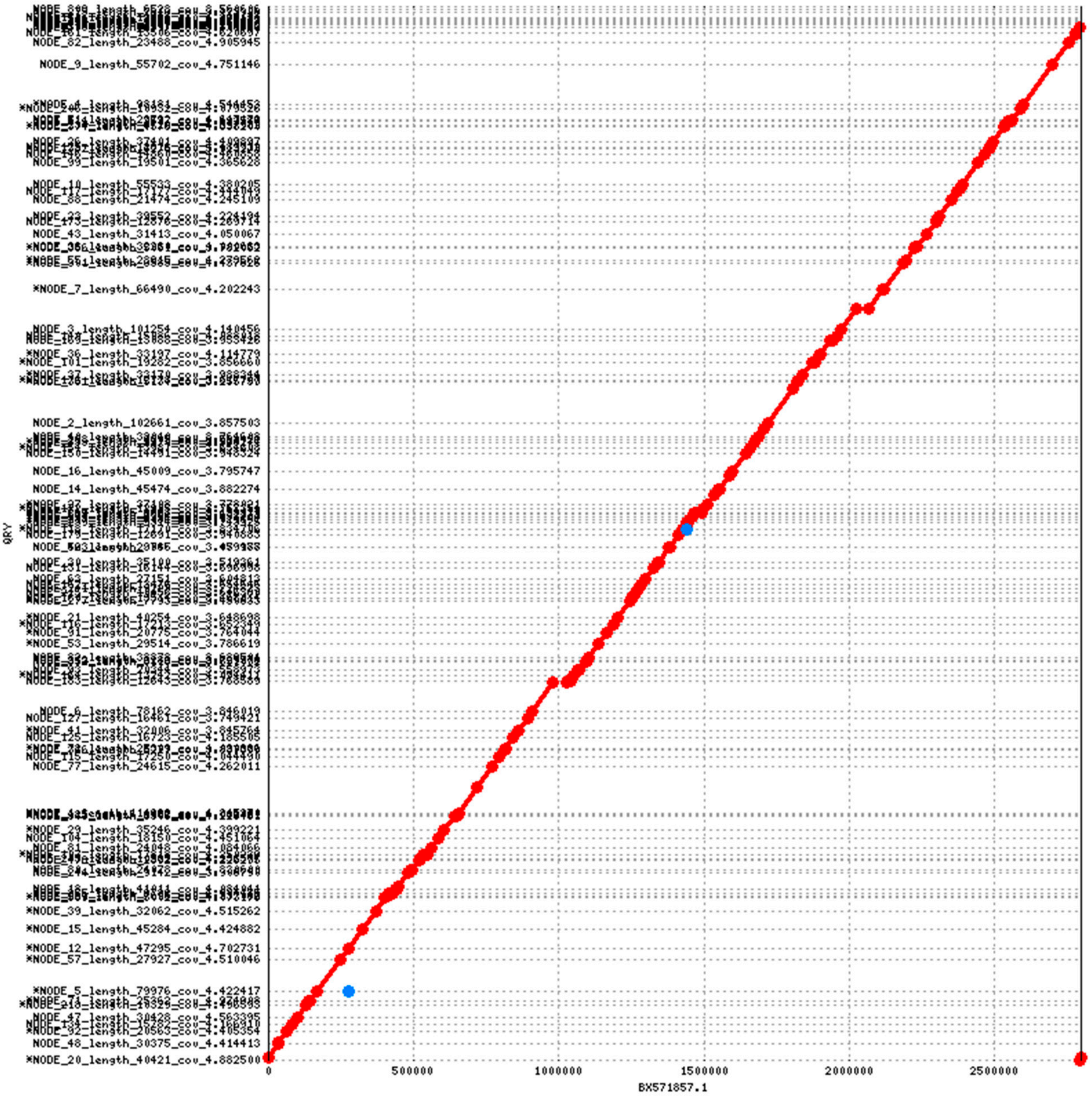

C. Mummer plot of MAG-1 onto reference sequence of *S. aureus* (BX571857) (sample from patient B).

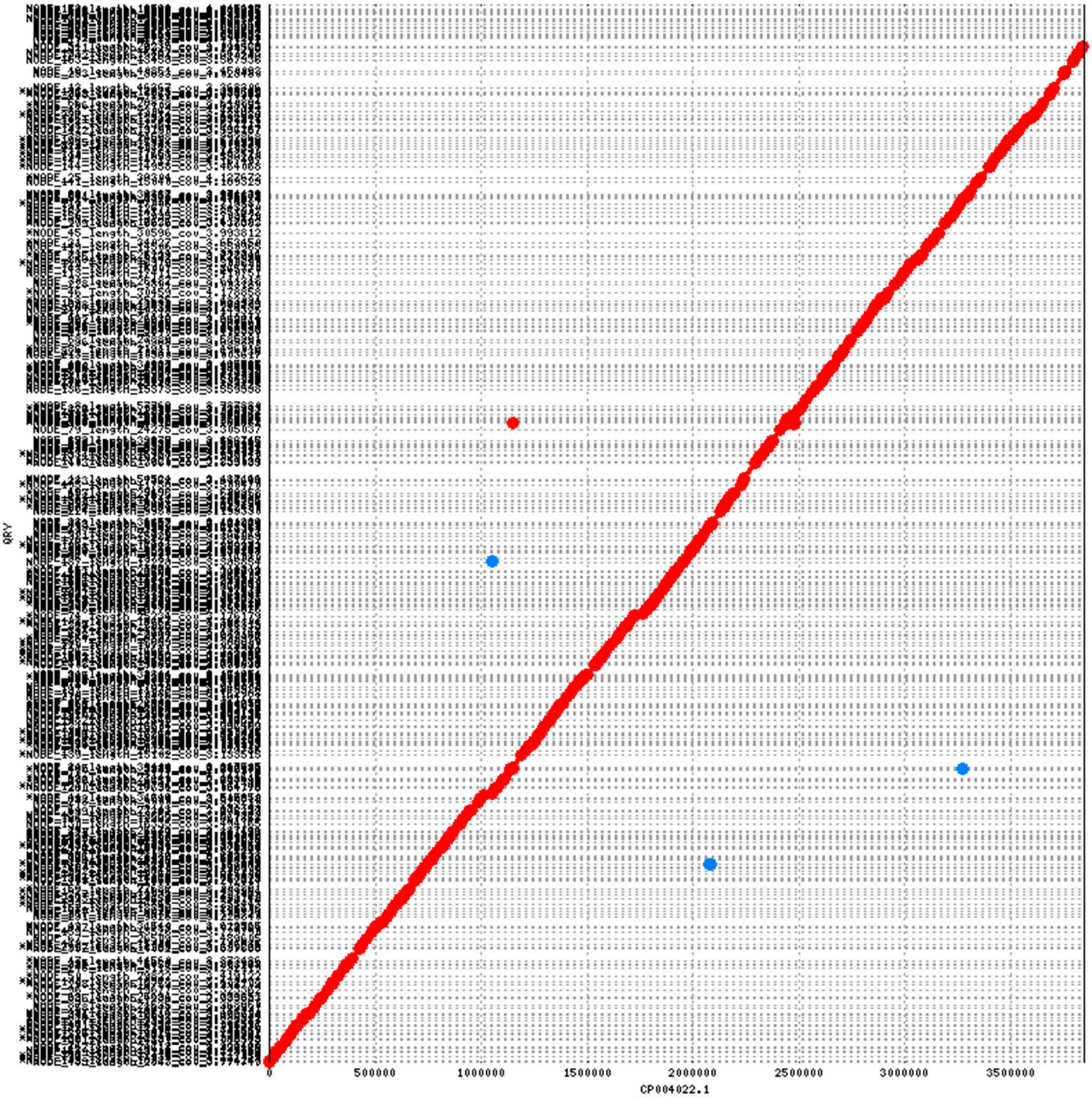

D. **Mummer plot of MAG-3 onto reference sequence of *Proteus mirablis* (CP004022_Proteus) (sample from Patient B).**

Table S1. The 38 contigs of the metagenome-assembled genome MAG-2 blast to *A. baumannii* genomes in the NCBI nucleotide database.

The *Moraxellaceae*-related metagenome-assembled genome (MAG-2) consisted of 38 contigs with 3,608 predicted genes based on metagenemark prediction. Similarity searches using BLAST to the NCBI nucleotide database revealed that the contigs were highly similar to *A. baumannii* J9 and *A. baumannii* 29FS20 (NZ_CP044519.1).

Contig ID	Best BLAST Hit
NODE_1_length_405957_cov_40.951757	NZ_CP041587.1 Acinetobacter baumannii strain J9
NODE_2_length_349529_cov_37.041806	NZ_CP041587.1 Acinetobacter baumannii strain J9
NODE_3_length_281707_cov_39.075689	NZ_CP041587.1 Acinetobacter baumannii strain J9
NODE_4_length_250614_cov_40.635323	NZ_CP041587.1 Acinetobacter baumannii strain J9
NODE_5_length_187596_cov_40.983038	NZ_CP041587.1 Acinetobacter baumannii strain J9
NODE_6_length_162015_cov_37.722913	NZ_CP041587.1 Acinetobacter baumannii strain J9
NODE_7_length_149223_cov_39.206318	NZ_CP051866.1 Acinetobacter baumannii strain Ab-C63
NODE_8_length_141783_cov_40.667398	NZ_CP041587.1 Acinetobacter baumannii strain J9
NODE_9_length_136449_cov_39.524085	NZ_CP041587.1 Acinetobacter baumannii strain J9
NODE_10_length_134270_cov_38.798525	NZ_CP041587.1 Acinetobacter baumannii strain J9
NODE_11_length_127909_cov_42.923913	NZ_CP041587.1 Acinetobacter baumannii strain J9
NODE_12_length_126774_cov_41.906739	NZ_CP041587.1 Acinetobacter baumannii strain J9
NODE_13_length_122587_cov_36.242010	NZ_LN997846.1 Acinetobacter baumannii isolate R2091 chromosome
NODE_14_length_110691_cov_37.655935	NZ_CP044517.1 Acinetobacter baumannii strain 31FS3-2
NODE_15_length_101105_cov_38.281831	NZ_CP041587.1 Acinetobacter baumannii strain J9
NODE_16_length_93769_cov_44.124677	NZ_CP044517.1 Acinetobacter baumannii strain 31FS3-2
NODE_17_length_93489_cov_39.960774	NZ_CP041587.1 Acinetobacter baumannii strain J9
NODE_18_length_93316_cov_38.022485	NZ_CP041587.1 Acinetobacter baumannii strain J9
NODE_19_length_79956_cov_33.971728	NZ_CP041587.1 Acinetobacter baumannii strain J9
NODE_20_length_76229_cov_46.640284	NZ_CP041587.1 Acinetobacter baumannii strain J9
NODE_21_length_74381_cov_33.527151	NZ_CP041587.1 Acinetobacter baumannii strain J9
NODE_22_length_64392_cov_39.959199	NZ_CP041587.1 Acinetobacter baumannii strain J9
NODE_23_length_56887_cov_37.444591	NZ_CP028138.1 Acinetobacter baumannii strain NCIMB 8209
NODE_26_length_50151_cov_45.245089	NZ_CP015364.1 Acinetobacter baumannii strain 3207
NODE_28_length_49607_cov_38.543772	NZ_CP041587.1 Acinetobacter baumannii strain J9
NODE_29_length_44144_cov_35.103813	NZ_CP041587.1 Acinetobacter baumannii strain J9
NODE_30_length_42841_cov_34.055250	NZ_CP033768.1 Acinetobacter baumannii strain FDAARGOS_533
NODE_31_length_42617_cov_40.423861	NZ_CP041587.1 Acinetobacter baumannii strain J9
NODE_33_length_41250_cov_39.263794	NZ_CP041587.1 Acinetobacter baumannii strain J9
NODE_34_length_38022_cov_41.065004	NZ_CP041587.1 Acinetobacter baumannii strain J9

NODE_37_length_31372_cov_38.336622	NZ_CP038503.1 Acinetobacter baumannii strain CIAT758 plasmid
NODE_47_length_26694_cov_39.004279	NZ_CP041587.1 Acinetobacter baumannii strain J9
NODE_105_length_12544_cov_37.228681	NZ_CP038258.1 Acinetobacter baumannii strain 39741
NODE_112_length_11798_cov_31.829090	NZ_CP041587.1 Acinetobacter baumannii strain J9
NODE_128_length_9951_cov_46.786681	NZ_CP041587.1 Acinetobacter baumannii strain J9
NODE_131_length_9800_cov_55.649051	NZ_CP041587.1 Acinetobacter baumannii strain J9
NODE_374_length_3940_cov_35.514028	NZ_CP041587.1 Acinetobacter baumannii strain J9
NODE_766_length_2549_cov_43.308340	NZ_CP047973.1 Acinetobacter baumannii strain DETAB-P2

*2.*Metabolic pathways analysis using KEGG pathways

Figure S3. Mapping of the genes and contigs to KEGG pathways.

(A) Metabolic pathway ‘Bacterial invasion’, (B) Metabolic pathway ‘Staphylococcus infection’ and (C) Metabolic pathway ‘Cationic antimicrobial peptides’. Only S. aureus pathway is shown. Color: green identified in patient A and B; orange: only identified in patient A; yellow: only identified in patient B; red: not identified in patient A and B

A.

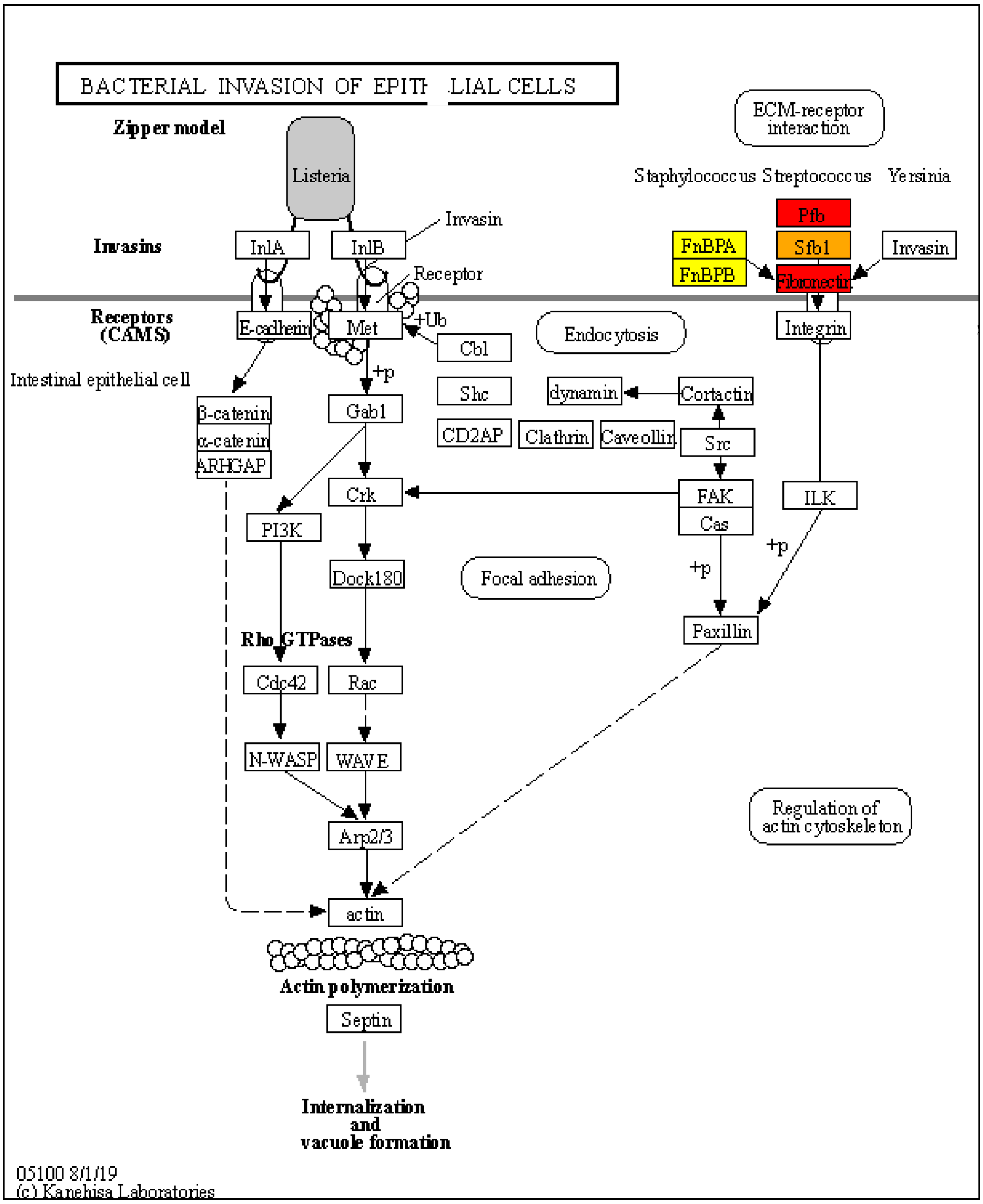
BACTERIAL INVASION OF EPITH LIAL CELLS
Zipper model
Listeria
ECM-receptor interaction
Staphylococcus
Streptococcus
Yersinia
Invasin
Invasins
InlA
InlB
FnBPA
FnBPB
Pfb
Sfb1
Fibronectin
Invasin
Receptor
Receptors (CAMS)
E-cadherin
Met
+Ub
Cbl
Endocytosis
Integrin
Intestinal epithelial cell
+p
β-catenin
α-catenin
ARHGAP
Gab1
Shc
dynamin
Cortactin
CD2AP
Clathrin
Caveollin
Src
Crk
FAK
Cas
ILK
PI3K
Dock180
Focal adhesion
+p
+p
Paxillin
Rho GTPases
Cdc42
Rac
N-WASP
WAVE
Arp2/3
Regulation of actin cytoskeleton
actin
Actin polymerization
Septin
Internalization and vacuole formation
05100 8/1/19
(c) Kanehisa Laboratories

B.

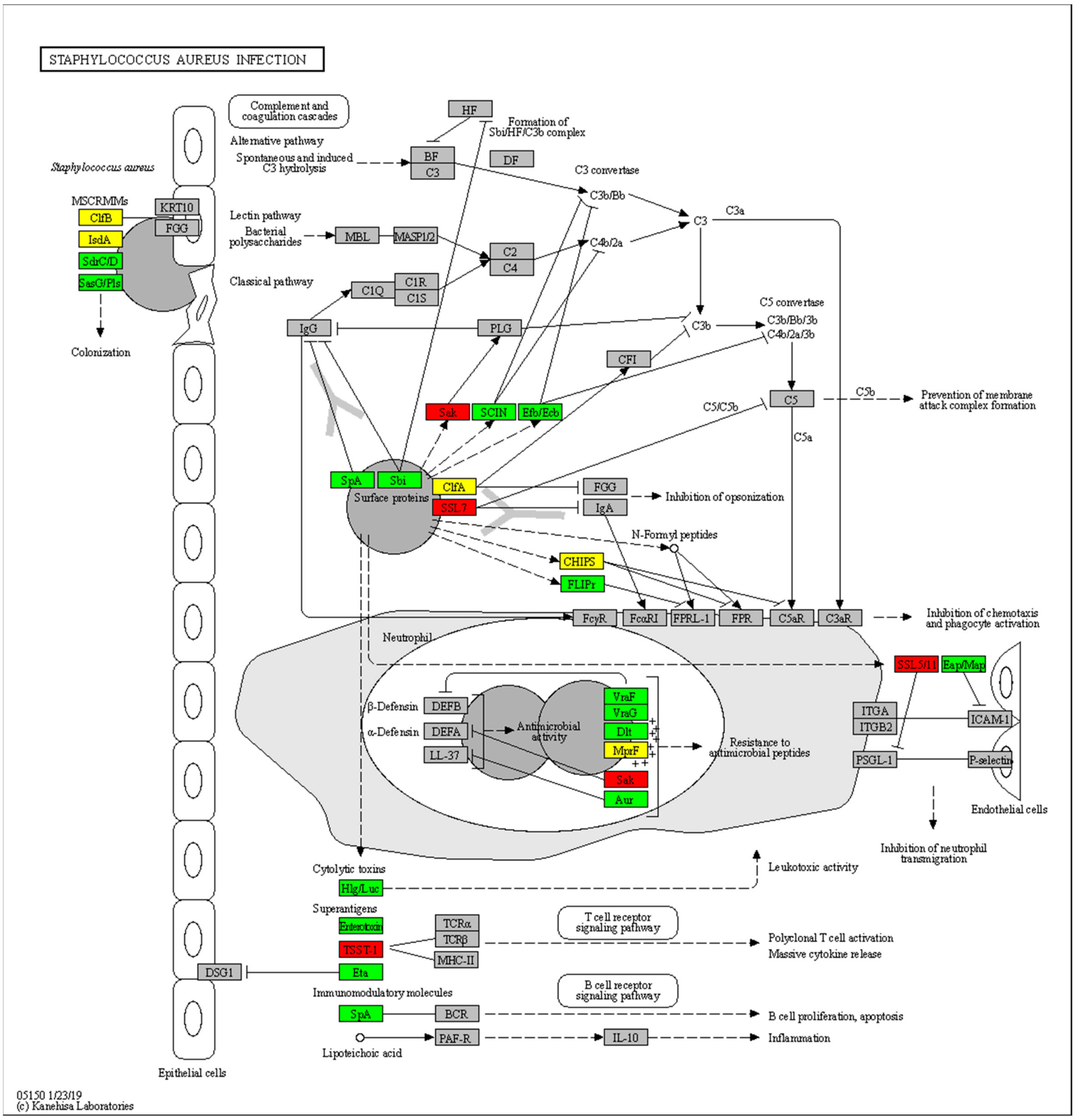
STAPHYLOCOCCUS AUREUS INFECTION
Staphylococcus aureus
MSCRMMs
ClfB
IsdA
SdrC/D
SasG/Pls
KRT10
FGG
Colonization
Complement and coagulation cascades
Alternative pathway
Spontaneous and induced C3 hydrolysis
HF
Formation of Sbi/HF/C3b complex
BF
C3
DF
C3 convertase
C3b/Bb
C3
C3a
Lectin pathway
Bacterial polysaccharides
MBL
MASP1/2
C2
C4
C4b/2a
Classical pathway
C1Q
C1R
C1S
IgG
PLG
C5 convertase
C3b
C3b/Bb/3b
C4b/2a/3b
CFI
C5/C5b
C5
C5b
Prevention of membrane attack complex formation
C5a
Sak
SCIN
Efb/Ecb
SpA
Sbi
Surface proteins
ClfA
SSL7
FGG
IgA
Inhibition of opsonization
N-Formyl peptides
CHIPS
FLIPr
FcγR
FcαRI
FPRL-1
FPR
C5aR
C3aR
Inhibition of chemotaxis and phagocyte activation
Neutrophil
SSL5/11
Eap/Map
β-Defensin
α-Defensin
DEFB
DEFA
LL-37
Antimicrobial activity
VraF
VraG
Dlt
MprF
Sak
Aur
Resistance to antimicrobial peptides
ITGA
ITGB2
ICAM-1
PSGL-1
P-selectin
Endothelial cells
Inhibition of neutrophil transmigration
Leukotoxic activity
Cytolytic toxins
Hlg/Luc
Superantigens
Enterotoxin
TSST-1
Eta
TCRα
TCRβ
MHC-II
T cell receptor signaling pathway
Polyclonal T cell activation
Massive cytokine release
DSG1
Immunomodulatory molecules
SpA
BCR
B cell receptor signaling pathway
B cell proliferation, apoptosis
Lipoteichoic acid
PAF-R
IL-10
Inflammation
Epithelial cells
05150 1/23/19
(c) Kanehisa Laboratories

C.

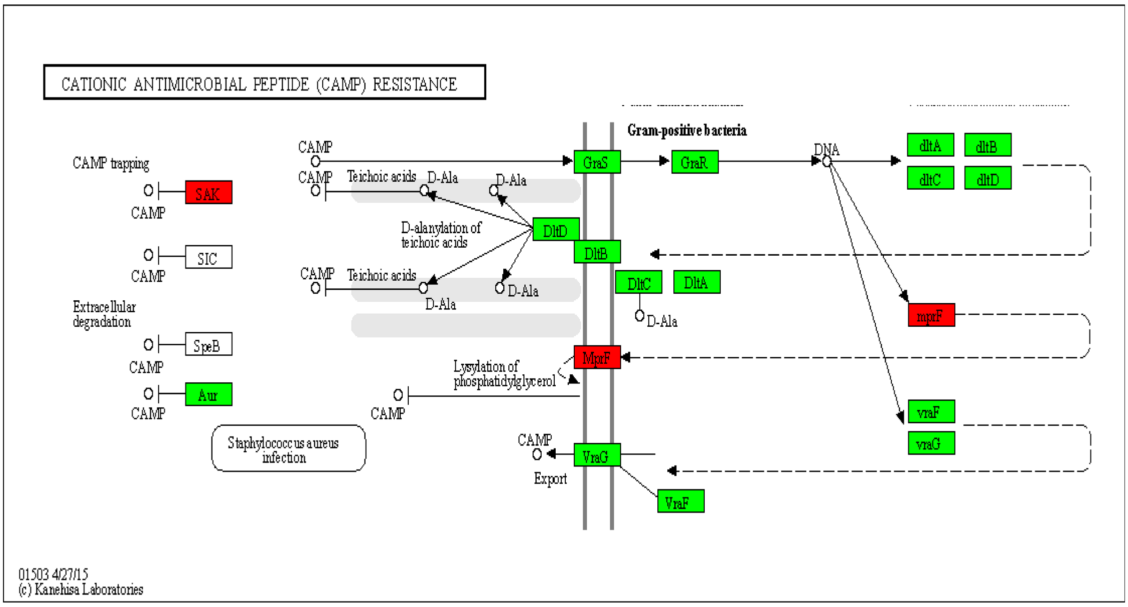
CATIONIC ANTIMICROBIAL PEPTIDE (CAMP) RESISTANCE
Gram-positive bacteria
CAMP trapping
SAK
CAMP
SIC
CAMP
Extracellular degradation
SpeB
CAMP
Aur
CAMP
Staphylococcus aureus infection
CAMP
GraS
GraR
DNA
dltA
dltB
dltC
dltD
CAMP
Teichoic acids
D-Ala
D-Ala
D-alanylation of teichoic acids
DltD
DltB
CAMP
Teichoic acids
D-Ala
D-Ala
DltC
DltA
D-Ala
mprF
MprF
Lysylation of phosphatidylglycerol
CAMP
vraF
vraG
CAMP
VraG
Export
VraF
01503 4/27/15
(c) Kanehisa Laboratories
